# Purpose-Driven Design: A Case Report of a Knowledge Mobilization Portal

**DOI:** 10.5334/pme.1791

**Published:** 2025-09-16

**Authors:** Deena M. Hamza, Anna MacLeod, Jonathan Sherbino, Anthony R. Artino, Kristina Dzara, Lauren A. Maggio, Robin Parker, Lara Varpio

**Affiliations:** 1 Office of Postgraduate Medical Education, Department of Medicine, University of Alberta, Canada; 2 Continuing Professional Development and Medical Education Research, Faculty of Medicine, Dalhousie University, Halifax, Canada; 3 McMaster Health Education Research, Innovation & Theory (MERIT) Centre, McMaster University, Hamilton, Canada; 4 Health, Human Function, and Rehabilitation Sciences, School of Medicine and Health Sciences, George Washington University, Washington, DC, USA; 5 Center for Educator Development, Advancement, and Research and Department of Family and Community Medicine, Saint Louis University School of Medicine, Saint Louis, MO, USA; 6 Department of Medical Education, University of Illinois Chicago, USA; 7 W.K. Kellogg Health Sciences Library, Dalhousie University, Canada; 8 Department of Pediatrics, Perelman School of Medicine at the University of Pennsylvania, The Children’s Hospital of Philadelphia, Philadelphia, PA, USA

## Abstract

**Background & Need for Innovation:**

Synthesizing academic literature is a foundational skill in health professions education (HPE), enabling evidence-informed decision-making and continuous improvement. However, privileging one review type as the “gold standard” reinforces a narrow hierarchy of evidence, marginalizing alternative worldviews and synthesis approaches.

**Goal of Innovation:**

This innovation aimed to broaden understanding and legitimate use of diverse literature synthesis methods by developing an accessible knowledge mobilization portal to support learners, educators, and scholars across the HPE community.

**Steps Taken for Development and Implementation of Innovation:**

We developed *LitR-Ex.com* *(Literature Reviews Explained)* to showcase eight literature synthesis methods—the Literature Review Series (LRS). The Eco-Normalization Framework guided the design, implementation, and reflexive evaluation of the portal, aligning the innovation with contextual affordances and user needs. A collaborative, values-driven approach supported content co-creation, informed by lived experience, mutual trust, and a shared commitment to inclusivity.

**Evaluation of Innovation:**

The platform successfully launched and has been sustained through a network of contributors. Informal feedback and web analytics suggest positive engagement, and early adopters report its utility in teaching and research contexts. The innovation’s resonance stems not only from its content but from the relationships and shared purpose underlying its development.

**Critical Reflection on Your Process:**

Key catalysts included: (1) friendship as an often-overlooked motivator in academic work; (2) trust and relationships that fostered momentum; and (3) a shared vision that anchored the innovation. These relational dimensions were as critical as the technical design in ensuring uptake and sustainability.

## Background and Need for the Innovation

The synthesis of academic literature in health professions education (HPE) is essential for evidence-informed decision-making, and for shaping the continuous quality improvement of curricula, programs, and everyday teaching and learning practices [1,2,3,4,5]. Knowledge syntheses, often disseminated as literature reviews, collate, summarize, integrate, and interpret existing knowledge. Given their foundational role, literature reviews must be the highest quality, upholding rigorous methodological practices and output expectations. Historically, however, HPE scholars have recognized a narrow scope of literature reviews as rigorous; such recognition has largely been reserved for syntheses steeped in the objectivist orientation (e.g., systematic reviews) and have been celebrated as rigorous because, when the methodology is appropriately followed, generalizability may be achieved [6]. Reviews that harness a subjectivist approach to research (e.g., narrative literature reviews like critical reviews and state-of-the-art reviews) were not well known with established methodologies. We suspect that this situation, paired with pressure to adhere to the positivist orientation, meant that subjectivist-oriented reviews were infrequently used in HPE [7]. As a result, HPE’s knowledge synthesis traditions had upheld a (tacit) evidence hierarchy that promoted the systematic review as the gold-standard, highest-quality, literature review method.

While HPE scholars have largely rejected the hierarchy that positions objectivist research as more rigorous, trustworthy, or “scientific” than subjectivist research [8], literature reviews steeped in a subjectivist orientation still struggle to be valued and widely adopted (see Table 1 for a summary of published review types guided by Barry et al., 2022; Maggio et al., 2021; and Thomas et al., 2020). Appreciating and using the full variety of literature reviews available for knowledge synthesis would enable HPE scholars to generate more diverse insights into HPE’s topics and problems. To ensure that a broad array of knowledge syntheses bolsters HPE’s academic literature, a literature review series (LRS) was developed and published in the *Journal of Graduate Medical Education (JGME)* [9]. In this series, eight different kinds of literature reviews were described, including explanations of each review’s paradigmatic orientations, purposes, methods, and markers of rigor.

**Table 1:** Literature review publications by type over the past 10 years.


PUBLICATION YEAR	CRITICAL	INTEGRATIVE	LITERATURE	META-ANALYSIS	NARRATIVE	QUALITATIVE	REALIST	REVIEW OF REVIEWS	SCOPING	STATE-OF-THE-ART	SYSTEMATIC	UMBRELLA

**2014**	2	1	8	4	2	0	0	0	1	0	19	0

**2015**	1	0	1	5	3	0	0	0	2	0	27	0

**2016**	3	0	12	5	4	0	0	0	3	0	31	1

**2017**	2	1	8	3	2	0	0	0	15	0	29	0

**2018**	1	2	10	3	6	0	0	0	12	0	26	0

**2019**	1	4	2	5	8	0	0	0	33	0	52	0

**2020**	1	3	5	6	4	0	0	0	36	0	32	0

**2021**	3	1	5	6	6	0	0	1	45	0	33	0

**2022**	4	1	7	7	13	0	0	0	78	2	60	1

**2023**	4	4	9	9	5	0	0	1	68	0	43	0

**2024**	1	3	12	20	14	0	0	0	80	2	47	1


However, changing the field’s perspective on the need to adopt a wider range of literature review types requires more than a series of papers explaining *how-to* and *why-to* use various literature review approaches. To influence the HPE academic community’s perspectives about the value and contributions offered in a wider array of literature reviews demanded a knowledge mobilization effort that reached across institutions and beyond one journal.

## Goal of the Innovation

**Figure 1:**
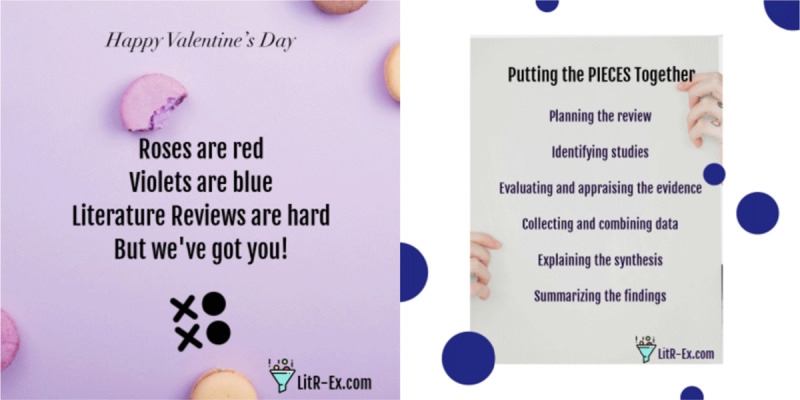
Example social media postings used to create awareness of the knowledge mobilization portal.

We sought to promote the adoption of a wider array of literature review methodologies across the HPE community. LitR-Ex.com (Literature Reviews Explained), hosted the LRS, offered additional resources (e.g., downloadable infographic guides) about each of the eight review types, and spotlighted the biographical sketches of all authors who contributed to the LRS. As such, LitR-Ex.com was designed with the following use-cases in mind: 1) Choosing review type – provide a broader array of knowledge synthesis methodologies such that scholars can select an appropriate method for their research question; 2) Accessing evidence-based rationale – provide scholars with theoretical grounding for each method; 3) Practical how-to guides – downloadable guides to aid in execution of each review type; and 4) Self-directed learning – a toolkit for all scholars to advance their knowledge, skills, and execution of diverse knowledge syntheses. The site was promoted via a social media strategy that leveraged purposefully created accounts for LitR-Ex on X, LinkedIn, Instagram, and Facebook, and the intentional amplification of these posts via re-posts and comments by HPE community champions. Figure 1 provides an example of social media postings used to create awareness of the knowledge mobilization portal and share brief strategies for conducting reviews.

## Steps Taken for Development and Implementation of Innovation

We used a purpose-centered approach to design, implement, and evaluate this innovation, informed by the Eco-Normalization Framework [10]. The Eco-Normalization Framework–recommended for shaping the successful design, implementation, and areas of inquiry for evaluation of an innovation [11] –prompts interest holders to consider the interactions between the purpose behind an innovation’s design and the cascading and interdependent effects of change on existing systems and people who are expected to enact or use the innovation. In alignment with this framework, we began by considering the purpose of our innovation (i.e., grand aspiration: having more types of literature reviews be used in HPE scholarship) in relation to the interconnections between the innovation (i.e., LitR-Ex.com), the people doing the work (i.e., the HPE scholars conducting literature reviews), and the systems in which the innovation is embedded (e.g., accepted norms of HPE scholars; peer-review journals’ expectations; values enacted in promotion and tenure decisions at individual academic institutions). Table 2 provides an abbreviated summary of these considerations, highlighting the relationship between JGME’s LRS and LitR-Ex.com and how these connections shaped the LitR-Ex.com innovation.

**Table 2:** Summary of reflections on purpose guided by the Eco-Normalization Framework.


SIX CORE AREAS OF INQUIRY	LITERATURE REVIEW SERIES (LRS)	LITR-EX.COM (MOBILIZING THE LRS)

*1. Grand Aspiration + Innovation(s)*	Broadening the methods used to make sense of rapidly growing literature to reflect the diversity of perspectives informing HPE and improve evidence-informed decision-making	Creating a series of resources to mobilize the LRS that can be accessed by the largest and broadest audience

*2. Grand Aspiration + People Doing the Work*	Advancing the methods of literature reviews in both theoretical and practical ways for immediate application	Establishing a user-centered hub for the literature review series that demonstrates utility and accessibility for use

*3. Grand Aspiration + System(s)*	Advancing the methods of literature reviews to accrue interest and investment from broad audiences, including journal editors, to increase the legitimacy of diverse approaches that also demonstrate rigor and credibility	Developing “at-a-glance” resources to support system-wide changes (e.g., reviewers can access resources to appraise literature reviews that diverge from mainstream approaches)

*4. Innovation + People Doing the Work*	Providing both philosophical/theoretical foundations and practical guides for researchers to get started on each review type and encourage diverse approaches	Providing all resources in one place that is user-centered and easy to use; creating space for the audience to draw their own conclusions about the best review type for their research question

*5. Innovation + System(s)*	Igniting reflection and potential changes to values, norms, and cultures regarding the “gold standard” review rhetoric through peer-reviewed publications	Creating a space with accessible and visually engaging content that includes published methods articles, summaries, and infographics to build momentum to transform the field

*6. System(s) + People Doing the Work*	Supporting and legitimizing the work of researchers through peer-reviewed methods publications to build collective action toward systems change	Launching a web and social media campaign to accrue interest, space, and investment in new ways of thinking about literature review methods to support and legitimize the work of researchers


**Figure 2:**
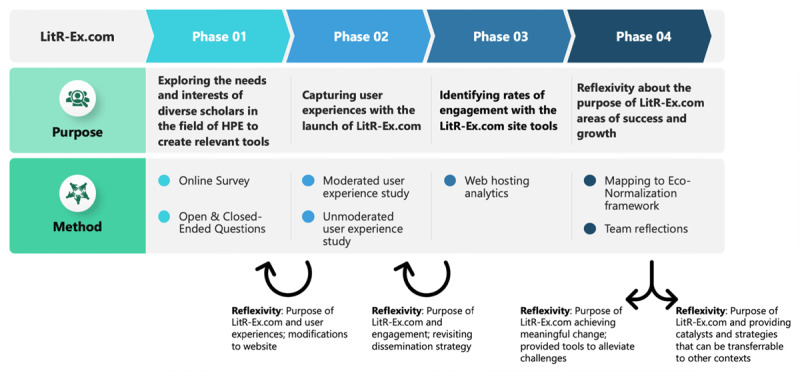
Purpose and Methods of Data Collection to Inform the LitR-Ex.com Innovation.

With these considerations in hand, we developed LitR-Ex.com by launching a four-phase, multi-methods data collection and analysis approach for continuously evaluating and iteratively refining the innovation (see Figure 2). This phased approach used reflexivity as an orientation for regularly revisiting the innovation’s progress in relation to all aspects of the Eco-Normalization Framework (i.e., grand aspiration; innovation; people doing the work; and systems), and having these insights inform the next phase of the innovation’s development. The data from Phase 1 informed the initial design of LitR-Ex.com, and data from later phases shaped its revision. Therefore, while we present evaluation data below by phase, notably LitR-Ex.com was developed using user input, and it has been regularly and iteratively examined and revised over the three years that it has been online (initially launched January 2022). The development and implementation of LitR-Ex.com has been continuous since its launch and continues today.

## Evaluation of Innovation

### Phase 1: Exploration Survey about Needs and Interests

The design of LitR-Ex.com was informed by Phase 1 data: an open and closed-ended online survey distributed through social media channels (Instagram, LinkedIn, X, and Facebook – these platforms were used for all project phases). We disseminated a survey to collect the needs and interests of diverse scholars related to conducting knowledge synthesis, and their previous experiences including resources they may have used when executing a knowledge synthesis (see Appendix A for participant information). Data from this initial survey (n = 95) were analyzed descriptively (closed-ended questions) and using manifest content analysis (open-ended questions). The survey was available from January 2022 – February 2022. The study was deemed exempt by the institutional research ethics board.

Participants reported that the most helpful elements of the resources they had historically accessed were step-by-step guides, reporting guidelines and examples, a clear delineation of the different types and purposes of literature reviews, descriptions of the process of conducting a literature review and synthesizing the data into a meaningful story, and librarian support. The least helpful aspects were resources that provided instructions that did not transfer across topics, online resources that lacked consistent conceptualizations of literature review types, absent foundational theory for review approaches and examples, and lack of knowledge and expertise of diverse literature review approaches.

These data confirmed the need for our innovation and shaped the development of the first version of LitR-Ex.com. They informed design decisions–e.g., to offer the innovation as a publicly available website, to create one-page how-to guides for each review type, to include direct links to the relevant LRS manuscript, to present summaries of pros-and-cons of the review type, to offer guidance about conducting literature reviews from academic librarians, and to highlight features that distinguish the review types apart from each other.

### Phase 2: Moderated and Unmoderated User Experience Studies

In Phase 2, moderated and unmoderated user experience studies captured data about the usability and accessibility of LitR-Ex.com, such as the ease of navigation of the knowledge mobilization portal and the utility of presented content. First, with a small sample of users (n = 3), the lead researcher (DMH) observed participants interact with LitR-Ex.com and collected data using a think-aloud protocol [12]. Data were then analyzed using manifest content analysis, and findings were used to re-design the knowledge mobilization portal. The participants (an early career librarian, an assistant professor with expertise in educational psychology and assessments, and a hospitalist clinician-educator with expertise in mixed methods medical education research) were prompted by the researcher to locate certain elements, which identified broken links and unintuitive button placements that needed redesign. Two participants suggested incorporating a decision tree approach to choosing the right review, while one participant noted that determining the right review is complex with innumerable permutations and combinations dependent on defining the problem and framing the research questions. Other suggestions focused on the aesthetics of the portal, streamlining the content to provide brief overviews followed by links to more elaborate descriptions, creating side-by-side comparisons of all review types, providing the timeline for the release of the next installment, and extracting strengths and weaknesses of each review type for ease of use. All participants endorsed the utility of the site content, particularly the how-to guides.

Second, unmoderated user experience surveys, specifically the system usability scale (SUS) [13] captured information about the portal’s effectiveness (e.g., facilitates achievement of objectives), efficiency (e.g., effort and resources required to achieve objectives), and user’s satisfaction (e.g., user’s experience) with the portal as a tool to support their literature review efforts. The survey available from April 2022–May 2024 was distributed on X, LinkedIn, Facebook, and Instagram via the LitR-Ex.com social media handle. The SUS comes with a specific scoring system from 0–100 (Note: a score of 68 is considered an average rating). It is the most frequently used instrument to measure usability of online products, such as websites [13].

A total of 20 participants provided information on the usability and accessibility of the LitR-Ex knowledge mobilization portal. According to their responses, the LitR-Ex.com portal scored 77.5 out of 100, suggesting a Grade B or Good rating for usability performance specific to effectiveness, efficiency, and overall ease of use. The survey also included questions about the most and least useful aspects of LitR-Ex.com and areas for improvement (see participant responses in Appendix B). These data were used to revise LitR-Ex.com with the most notable changes being the revised layout within page navigation, brief side-by-side review type comparison, reduction of extraneous images and refinement of summaries, a distinct strengths and weaknesses section, and simple and improved navigation.

### Phase 3: Engagement Measured through Web Analytics

Since its launch in January 2022, LitR-Ex.com has been accessed by 6,201 unique users from 123 countries. Appendix C illustrates the number of unique visitors for each of the 123 countries with the United States, Canada, the United Kingdom, Australia, and India having the highest number of visitors to the site from January 2022–January 2025.

### Phase 4: Reflexive Interview – Bringing It Back to Purpose

Finally, Phase 4 involved a 60-minute reflective dialogue with core team members (LV, AM, RP). One researcher (DMH) used the Eco-Normalization Framework to guide the discussion and capture the genesis, purpose, and processes involved to generate and launch the LRS. The dialogue explored the mobilization of LitR-Ex.com and the considerations that enabled and impeded success. The transcript was analyzed using reflexive thematic analysis [14] and findings were shared with the core (LV, AM, RP) and broader LitR-Ex.com team (AA, KD, LM, JS) to comment on data interpretations and to supplement content where needed (member reflection).

## Critical Reflection on the Process

These findings generated five key catalysts relevant to others endeavoring to launch knowledge mobilization innovations.

### 1. Friendships

Core team members (LV, AM, RP) reflected on how their friendship and past positive collaboration experiences fertilized an innovation. Friendships are an often-unreported aspect of innovation and collaboration and continue to be absent in academic writing [15] yet are meaningful and illuminating discourses. An initial, between-friends conversation rapidly evolved into a purpose and mission to transform the ways in which HPE scholars think about, conceptualize, and enact knowledge syntheses. This led to the LRS and then to LitR-Ex.com.

### 2. The Network

The team members highlighted the importance of their shared network of names of trusted colleagues, each of whom held specific skills and expertise in a specific literature review method. The complementarity of skills of each team member, the foundation of trusted colleagues, and the idea that everyone could equally contribute to the LRS (i.e., no one “needed to be the hero of the story”) nurtured the momentum necessary to garner interest and investment from invited collaborators to contribute to the LRS as well as the LitR-Ex.com mobilization effort.

### 3. The Ballast

Each of the eight literature review methods included an author who is referred to as the “ballast” – i.e., a person on whom the editors could rely on to ensure the assigned work was completed and was completed *well*. Returning to the theme of relationships, these were individuals who could navigate the roles of distributed responsibility and leadership to cultivate and make manageable a complex innovation.

### 4. Uplifting Others

While the initial momentum for this innovation came from known networks and established scholars, the editorial team and all invited collaborators shared a goal of uplifting early career researchers and under-represented scholars through the LRS. They viewed the LRS and LitR-Ex.com as vehicles to contribute to this social uplift agenda. They decided that each publication would include an early-to-mid career researcher whose career could be enhanced through these innovations and whose perspectives would enhance the process and end product. These authors were highlighted via bio sketches on LitR-Ex.com and promoted via the social media campaign.

### 5. Coherence

Transforming the ways in which the HPE community thinks about literature reviews requires cognitive participation to cultivate a movement toward change. A significant factor that facilitated the launch of LitR-Ex.com was a shared mental model, or a mental conceptual framework in one’s mind that guided decision-making [16], informed by a common goal described in Table 2, which allowed the complexities of multiple parts of the innovation with multiple distributed leaders ensure that the portal was coherent in the design and delivery.

## Conclusion

The LRS and LitR-Ex.com represent a step forward in advancing scholarship in HPE by broadening the scope and acceptability of a diversity of literature reviews. Grounded in a strong theoretical foundation, these innovations provide practical resources that support more diverse and sophisticated synthesis methods. Historically, systematic and scoping reviews have dominated the field [7] but recent trends indicate a growing recognition of other review types that offer nuanced insights and broader applicability. Notably, no integrative reviews were published in five core HPE journals in 2020, yet following the launch of LRS and LitR-Ex.com, three have since been published (Appendix D) [17,18,19]. By mobilizing knowledge and fostering engagement among journal editors, authors, and peer reviewers, we seek to cultivate a more nuanced and integrative approach to literature synthesis. This shift holds great promise for shaping the future of HPE research, ensuring that literature reviews evolve to be not only rigorous but also generative, inclusive, and transformative. LitR-Ex.com exemplifies how purpose-driven design can transform the field by bridging the gap between research and real-world application. By actively disseminating advancement in HPE in an accessible, practice-oriented format, it fosters evidence-informed decision-making among educators, scientists, researchers, and clinicians. By leveraging technology and design thinking, educators now have a robust process model to create more responsive and learner-centered training environments.

## Additional File

The additional file for this article can be found as follows:

10.5334/pme.1791.s1 Appendices. Appendix A to D.
