# Supplementary material for: Purpose-Driven Design: A Case Report of a Knowledge Mobilization Portal
Source: Perspect Med Educ. 2025 Sep 16;14(1):570–7. doi: 10.5334/pme.1791 (PMC12447794; doi:10.5334/pme.1791)
Supplement: Appendices. — Appendix A to D. [file pme-14-1-1791-s1.pdf]

APPENDIX A. Needs assessment data from 95 participants to help inform the development of LitR-Ex.com

Additional contextual information from the needs assessment
• 81% (n=48) indicated that they have led or participated in a literature review in the past<br>• 79% (n= 75) indicated that they had plans to conduct a literature review, with 65% (n=29) reporting a start date within the next six months<br>• 58% (n=25) reported intending to conduct a scoping review<br>• 37% (n=16) planning a systematic review<br>• 35% (n=15) planning a narrative review<br>• A small percentage of participants indicated their plan to conduct an umbrella review (5%; n=2); an integrative review or mapping review (7%; n=3); and a realist review (12%; n=5).

APPENDIX B Data from the unmoderated user experience study (n=20) describing aspects of LitR-Ex.com

**The most useful aspects of LitR-Ex.com**	**The least useful aspects of LitR-Ex.com**
• Brief overviews of each of the eight literature review types<br>• Links to the full publications<br>• Common layout of each page of the website<br>• Librarian recommendations section<br>• Detailed descriptions of how and when to use the diverse review approaches	• Headings that lacked contrast with the background image<br>• The additional resources section<br>• The genesis of the literature review series was appreciated but reported to be too long<br>• Pending news and events page that was still under development<br>• A section about if one really needs to conduct a literature review.
**Suggestions for Improvement**	
• Curation of a reporting guidelines section<br>• Examples of published reviews for each of the approaches<br>• An at-a-glance one-page overview of all reviews that connects to more detailed sections<br>• Removal of excessive pictures throughout the website<br>• Removal of the “learn more” buttons throughout the website and reduce constant scrolling<br>• Ensuring text is left-justified<br>• Modifying the tone of the About page to be less colloquial<br>• Creating a counter example, such as a different method to answer the research question(s), as a separate section that informs if one really needs to conduct a literature review	

APPENDIX C. Web analytics accessing the LitR-Ex.com site from January 2022 – January 2025

Country Code	Country Name	Unique Visitors
US	United States	1814
CA	Canada	1590
GB	United Kingdom	686
AU	Australia	355
IN	India	149
DE	Germany	138
PT	Portugal	116
NL	Netherlands	112
IE	Ireland	101
RU	Russia	77
ES	Spain	71
ZA	South Africa	65
BR	Brazil	63
SE	Sweden	62
PH	Philippines	60
IT	Italy	53
FR	France	53
CN	China	49
CO	Colombia	48
SG	Singapore	45
DK	Denmark	45
SA	Saudi Arabia	40
MX	Mexico	40
CH	Switzerland	36
PK	Pakistan	35
FI	Finland	34
ID	Indonesia	32
NO	Norway	30
BE	Belgium	30
NZ	New Zealand	29
PL	Poland	27
MY	Malaysia	26
JP	Japan	25
AT	Austria	25
TR	Turkey	24
EG	Egypt	24
TH	Thailand	24

GR	Greece	22
CL	Chile	22
AE	United Arab Emirates	21
PE	Peru	21
HK	Hong Kong	19
TW	Taiwan	17
ET	Ethiopia	17
OM	Oman	17
NG	Nigeria	17
IR	Iran	15
KR	South Korea	15
RO	Romania	12
QA	Qatar	12
IQ	Iraq	11
CZ	Czech Republic	11
IL	Israel	11
MA	Morocco	11
VN	Vietnam	10
KE	Kenya	8
JO	Jordan	8
UG	Uganda	7
TZ	Tanzania	7
KW	Kuwait	7
ZW	Zimbabwe	7
ZM	Zambia	6
GH	Ghana	6
EC	Ecuador	6
BN	Brunei	6
FJ	Fiji	6
AR	Argentina	5
LB	Lebanon	5
GD	Grenada	4
NP	Nepal	4
BH	Bahrain	4
JM	Jamaica	4
BG	Bulgaria	4
DZ	Algeria	4
LV	Latvia	4
LT	Lithuania	4
LK	Sri Lanka	4
VE	Venezuela	3
RS	Serbia	3
AM	Armenia	3
MW	Malawi	3
HR	Croatia	3

PR	Puerto Rico	3
NA	Namibia	3
UZ	Uzbekistan	3
KZ	Kazakhstan	3
HU	Hungary	2
KG	Kyrgyzstan	2
BD	Bangladesh	2
KY	Cayman Islands	2
CY	Cyprus	2
MV	Maldives	2
MT	Malta	2
BA	Bosnia and Herzegovina	2
EE	Estonia	2
MU	Mauritius	2
UA	Ukraine	2
LC	Saint Lucia	1
TT	Trinidad and Tobago	1
LY	Libya	1
GI	Gibraltar	1
GT	Guatemala	1
AZ	Azerbaijan	1
LU	Luxembourg	1
AD	Andorra	1
MK	North Macedonia	1
IS	Iceland	1
LA	Laos	1
KH	Cambodia	1
BI	Burundi	1
BJ	Benin	1
IM	Isle of Man	1
GG	Guernsey	1
AF	Afghanistan	1
SZ	Eswatini	1
UY	Uruguay	1
SV	El Salvador	1
Unknown	Unknown	1
SD	Sudan	1
SI	Slovenia	1
DO	Dominican Republic	1
GE	Georgia	1
GY	Guyana	1
XK	Kosovo	1
BO	Bolivia	1
SS	South Sudan	1

PY	Paraguay	1
YE	Yemen	1
**Total**	**-**	**6201**

Appendix D Early signals of moving the field: literature review counts by type comparing those published in 2020 with those publish in 2024 in five core health professions education journals.

Year	Citation
**2020**	**Medical Education**
	**Systematic Review (N=9)**
	Clapper TC, Ching K. Debunking the myth that the majority of medical errors are attributed to communication. *Med Educ*. 2020; 54: 74–81. https://doi.org/10.1111/medu.13821
	Hancock J, Mattick K. Tolerance of ambiguity and psychological well-being in medical training: A systematic review. *Med Educ*. 2020; 54: 125–137. https://doi.org/10.1111/medu.14031
	Scheepers RA, Emke H, Epstein RM, Lombarts KMJMH. The impact of mindfulness-based interventions on doctors' well-being and performance: A systematic review. *Med Educ*. 2020; 54: 138-149. https://doi.org/10.1111/medu.14020
	Brennan N, Price T, Archer J, Brett J. Remediating professionalism lapses in medical students and doctors: A systematic review. *Med Educ*. 2020; 54: 196–204. https://doi.org/10.1111/medu.14016
	Dalwood N, Bowles K-A, Williams C, Morgan P, Pritchard S, Blackstock F. Students as patients: A systematic review of peer simulation in health care professional education. *Med Educ*. 2020; 54: 387–399. https://doi.org/10.1111/medu.14058
	Noyes JA, Welch PM, Johnson JW, Carbonneau KJ. A systematic review of digital badges in health care education. *Med Educ*. 2020; 54: 600–615. https://doi.org/10.1111/medu.14060
	Piot M-A, Dechartres A, Attoe C, et al. Simulation in psychiatry for medical doctors: A systematic review and meta-analysis. *Med Educ*. 2020; 54: 696–708. https://doi.org/10.1111/medu.14166
	Lee J, Kim H, Kim KH, Jung D, Jowsey T, Webster CS. Effective virtual patient simulators for medical communication training: A systematic review. *Med Educ*. 2020; 54: 786–795. https://doi.org/10.1111/medu.14152
	Webster ES, Paton LW, Crampton PES, Tiffin PA. Situational judgement test validity for selection: A systematic review and meta-analysis. *Med Educ*. 2020; 54: 888-902. https://doi.org/10.1111/medu.14201
	**Narrative Review (N=1)**
	Martimianakis MA (Tina), Tilburt J, Michalec B, Hafferty FW. Myths and social structure: The unbearable necessity of mythology in medical education. *Med Educ*. 2020; 54: 15–21. https://doi.org/10.1111/medu.13828
	**Critical Review (N=2)**
	Jauregui J, Watsjold B, Welsh L, Ilgen JS, Robins L. Generational "othering": The myth of the Millennial learner. *Med Educ*. 2020; 54: 60–65. https://doi.org/10.1111/medu.13795
	McGrath C, Liljedahl M, Palmgren PJ. You say it, we say it, but how do we use it? Communities of practice: A critical analysis. *Med Educ*. 2020; 54: 188–195. https://doi.org/10.1111/medu.14021
	**State-of-the-art Review (N=0)**
	**Realist Reviews (N=1)**
	Richmond A, Cooper N, Gay S, Atiomo W, Patel R. The student is key: A realist review of educational interventions to develop analytical and non-analytical clinical reasoning ability. *Med Educ*. 2020; 54: 709–719. https://doi.org/10.1111/medu.14137
	**Scoping Review (N=4)**
	Gottlieb M, Chung A, Battaglioli N, Sebok-Syer SS, Kalantari A. Impostor syndrome among physicians and physicians in training: A scoping review. *Med Educ*. 2020; 54: 116–124. https://doi.org/10.1111/medu.13956
	Versteeg M, Hendriks RA, Thomas A, Ommering BWC, Steendijk P. Conceptualising spaced

	learning in health professions education: A scoping review. *Med Educ*. 2020; 54: 205–216. https://doi.org/10.1111/medu.14025
	Crowther LL, Roberston N, Anderson ES. Mindfulness for undergraduate health and social care professional students: Findings from a qualitative scoping review using the 3P model. *Med Educ*. 2020; 54: 796–810. https://doi.org/10.1111/medu.14150
	St-Onge C, Vachon Lachiver É, Langevin S, Boileau E, Bernier F, Thomas A. Lessons from the implementation of developmental progress assessment: A scoping review. *Med Educ*. 2020; 54: 878–887. https://doi.org/10.1111/medu.14136
	**Meta-Ethnographic Reviews (N=0)**
	**Integrative Reviews (N=0)**
	**Academic Medicine**
	**Systematic Review (N=7)**
	Kaltman, Stacey PhD; Tankersley, Amelia MS. Teaching Motivational Interviewing to Medical Students: A Systematic Review. Academic Medicine 95(3):p 458-469, March 2020. | DOI: 10.1097/ACM.0000000000003011
	Scott, Michael D. MD, FRCPC; McQueen, Sydney MSc; Richardson, Lisa MD, MA, FRCPC. Teaching Health Advocacy: A Systematic Review of Educational Interventions for Postgraduate Medical Trainees. Academic Medicine 95(4):p 644-656, April 2020. | DOI: 10.1097/ACM.0000000000003063
	Berger, Arielle S. MD; Niedra, Elizabeth MD; Brooks, Stephanie G.; Ahmed, Waleed S. MD; Ginsburg, Shiphra MD, PhD. Teaching Professionalism in Postgraduate Medical Education: A Systematic Review. Academic Medicine 95(6):p 938-946, June 2020. | DOI: 10.1097/ACM.0000000000002987
	Fragkos, Konstantinos C. MBBS, MSc, MA, PhD; Crampton, Paul E.S. MSc, PhD. The Effectiveness of Teaching Clinical Empathy to Medical Students: A Systematic Review and Meta-Analysis of Randomized Controlled Trials. Academic Medicine 95(6):p 947-957, June 2020. | DOI: 10.1097/ACM.0000000000003058
	Zaorsky, Nicholas G. MD; O'Brien, Emily; Mardini, Jennifer; Lehrer, Eric J. MD, MS; Holliday, Emma MD; Weisman, Carol S. PhD. Publication Productivity and Academic Rank in Medicine: A Systematic Review and Meta-Analysis. Academic Medicine 95(8):p 1274-1282, August 2020. | DOI: 10.1097/ACM.0000000000003185
	Prentice, Shaun; Dorstyn, Diana MPsych (Clin), PhD; Benson, Jill MBBS, PhD, FACRRM; Elliott, Taryn PhD. Burnout Levels and Patterns in Postgraduate Medical Trainees: A Systematic Review and Meta-Analysis. Academic Medicine 95(9):p 1444-1454, September 2020. | DOI: 10.1097/ACM.0000000000003379
	Todd, Anna R. MD, PhD; Cawthorn, Thomas R. MD, MSc; Temple-Oberle, Claire MD, MSc, FRCSC. Pregnancy and Parenthood Remain Challenging During Surgical Residency: A Systematic Review. Academic Medicine 95(10):p 1607-1615, October 2020. | DOI: 10.1097/ACM.0000000000003351
	**Narrative Review (N=1)**
	Barber, Cassandra MA; van der Vleuten, Cees PhD; Leppink, Jimmie PhD; Chahine, Saad PhD. Social Accountability Frameworks and Their Implications for Medical Education and Program Evaluation: A Narrative Review. Academic Medicine 95(12):p 1945-1954, December 2020. | DOI: 10.1097/ACM.0000000000003731
	**Critical Review (N=0)**
	**State-of-the-art Review (N=0)**
	**Realist Reviews (N=0)**
	**Scoping Review (N=6)**
	Helou, Marieka A. MD, MPH; DiazGranados, Deborah PhD, MS; Ryan, Michael S. MD, MEHP; Cyrus, John W. MLIS. Uncertainty in Decision Making in Medicine: A Scoping Review and Thematic Analysis of Conceptual Models. Academic Medicine 95(1):p 157-165, January 2020. | DOI: 10.1097/ACM.0000000000002902
	Lewis, Lerona Dana PhD; Steinert, Yvonne PhD. How Culture Is Understood in Faculty Development

	in the Health Professions: A Scoping Review. Academic Medicine 95(2):p 310-319, February 2020. | DOI: 10.1097/ACM.0000000000003024
	Muzyk, Andrew PharmD, MHPE; Smothers, Zachary P.W. MS; Andolsek, Kathryn M. MD, MPH; Bradner, Melissa MD, MSHA; Bratberg, Jeffrey P. PharmD; Clark, Seth A. MD, MPH; Collins, Kathryn; Greskovic, Gerard A. BSPharm; Gruppen, Larry PhD; MacEachern, Mark MLIS; Ramsey, Susan E. PhD; Ruiz Veve, Jennifer; Tetrault, Jeanette M. MD. Interprofessional Substance Use Disorder Education in Health Professions Education Programs: A Scoping Review. Academic Medicine 95(3):p 470-480, March 2020. | DOI: 10.1097/ACM.0000000000003053
	Brottman, Melissa R. OTR/L, OTD; Char, Douglas M. MD, MA; Hattori, Robin A. MA; Heeb, Rachel OTR/L, OTD; Taff, Steven D. PhD, OTR/L. Toward Cultural Competency in Health Care: A Scoping Review of the Diversity and Inclusion Education Literature. Academic Medicine 95(5):p 803-813, May 2020. | DOI: 10.1097/ACM.0000000000002995
	Manca, Annalisa MEd; Gormley, Gerard J. MD, FRCGP; Johnston, Jennifer L. PhD, MRCGP; Hart, Nigel D. MD, MMedSc, FRCGP. Honoring Medicine's Social Contract: A Scoping Review of Critical Consciousness in Medical Education. Academic Medicine 95(6):p 958-967, June 2020. | DOI: 10.1097/ACM.0000000000003059
	Lochnan, Heather MD, FRCPC; Kitto, Simon PhD; Danilovich, Natalia MD, MSc, PhD; Viner, Gary MD; Walsh, Allyn MD, CCFP; Oandasan, Ivy F. MD, CCFP, MHSc; Hendry, Paul MD, MSc, FRCSC. Conceptualization of Competency-Based Medical Education Terminology in Family Medicine Postgraduate Medical Education and Continuing Professional Development: A Scoping Review. Academic Medicine 95(7):p 1106-1119, July 2020. | DOI: 10.1097/ACM.0000000000003178
	**Meta-Ethnographic Reviews (N=0)**
	**Integrative Reviews (N=0)**
	**Medical Teacher**
	**Systematic Review (N=6)**
	Gordon, M., Gupta, S., Thornton, D., Reid, M., Mallen, E., & Melling, A. (2019). Patient/service user involvement in medical education: A best evidence medical education (BEME) systematic review: BEME Guide No. 58. *Medical Teacher*, *42*(1), 4–16. https://doi.org/10.1080/0142159X.2019.1652731
	Barnes, T., Yu, T. C. W., & Webster, C. S. (2019). Preparedness of medical students and junior doctors for their role as clinical leaders: A systematic review. *Medical Teacher*, *42*(1), 79–85. https://doi.org/10.1080/0142159X.2019.1665632
	Mortaz Hejri, S., Jalili, M., Masoomi, R., Shirazi, M., Nedjat, S., & Norcini, J. (2019). The utility of mini-Clinical Evaluation Exercise in undergraduate and postgraduate medical education: A BEME review: BEME Guide No. 59 . *Medical Teacher*, *42*(2), 125–142. https://doi.org/10.1080/0142159X.2019.1652732
	Huey, C. W. T., & Palaganas, J. C. (2020). What are the factors affecting resilience in health professionals? A synthesis of systematic reviews. *Medical Teacher*, *42*(5), 550–560. https://doi.org/10.1080/0142159X.2020.1714020
	Ashokka, B., Dong, C., Law, L. S. C., Liaw, S. Y., Chen, F. G., & Samarasekera, D. D. (2020). A BEME systematic review of teaching interventions to equip medical students and residents in early recognition and prompt escalation of acute clinical deteriorations: BEME Guide No. 62. *Medical Teacher*, *42*(7), 724–737. https://doi.org/10.1080/0142159X.2020.1763286
	Gordon, M., Patricio, M., Horne, L., Muston, A., Alston, S. R., Pammi, M., … Daniel, M. (2020). Developments in medical education in response to the COVID-19 pandemic: A rapid BEME systematic review: BEME Guide No. 63. *Medical Teacher*, *42*(11), 1202–1215. https://doi.org/10.1080/0142159X.2020.1807484
	**Narrative Review (N=1)**
	Srinivasa, K., Chen, Y., & Henning, M. A. (2020). The role of online videos in teaching procedural skills to post-graduate medical learners: A systematic narrative review. *Medical Teacher*, *42*(6), 689–697. https://doi.org/10.1080/0142159X.2020.1733507
	**Critical Review (N=0)**
	**State-of-the-art Review (N=0)**
	**Realist Reviews (N=1)**

	de Groot, E., Schönrock-Adema, J., Zwart, D., Damoiseaux, R., Van den Bogerd, K., Diemers, A., … Bombeke, K. (2019). Learning from patients about patient-centeredness: A realist review: BEME Guide No. 60. *Medical Teacher*, *42*(4), 380–392. https://doi.org/10.1080/0142159X.2019.1695767
	Pierce, C., Corral, J., Aagaard, E., Harnke, B., Irby, D. M., & Stickrath, C. (2020). A BEME realist synthesis review of the effectiveness of teaching strategies used in the clinical setting on the development of clinical skills among health professionals: BEME Guide No. 61. *Medical Teacher*, *42*(6), 604–615. https://doi.org/10.1080/0142159X.2019.1708294
	**Scoping Review (N=5)**
	Pagatpatan, C. P., Valdezco, J. A. T., & Lauron, J. D. C. (2020). Teaching the affective domain in community-based medical education: A scoping review. *Medical Teacher*, *42*(5), 507–514. https://doi.org/10.1080/0142159X.2019.1707175
	Ong, Y. T., Kow, C. S., Teo, Y. H., Tan, L. H. E., Abdurrahman, A. B. H. M., Quek, N. W. S., Radha Krishna, L. K. (2020). Nurturing professionalism in medical schools. A systematic scoping review of training curricula between 1990–2019. *Medical Teacher*, *42*(6), 636–649. https://doi.org/10.1080/0142159X.2020.1724921
	Sirianni, G., Glover Takahashi, S., & Myers, J. (2020). Taking stock of what is known about faculty development in competency-based medical education: A scoping review paper. *Medical Teacher*, *42*(8), 909–915. https://doi.org/10.1080/0142159X.2020.1763285
	Stovel, R. G., Gabarin, N., Cavalcanti, R. B., & Abrams, H. (2020). Curricular needs for training telemedicine physicians: A scoping review. *Medical Teacher*, *42*(11), 1234–1242. https://doi.org/10.1080/0142159X.2020.1799959
	Jouda, M., & Finn, Y. (2020). Training in polytrauma management in medical curricula: A scoping review. *Medical Teacher*, *42*(12), 1385–1393. https://doi.org/10.1080/0142159X.2020.1811845
	**Meta-Ethnographic Reviews (N=0)**
	**Integrative Reviews (N=0)**
	**Advances in Health Science Education**
	**Systematic Review (N=0)**
	**Narrative Review (N=0)**
	**Critical Review (N=2)**
	Kahlke, R.M., McConnell, M.M., Wisener, K.M. *et al.* The disconnect between knowing and doing in health professions education and practice. *Adv in Health Sci Educ* **25**, 227–240 (2020). https://doi.org/10.1007/s10459-019-09886-5
	Tolsgaard, M.G., Boscardin, C.K., Park, Y.S. *et al.* The role of data science and machine learning in Health Professions Education: practical applications, theoretical contributions, and epistemic beliefs. *Adv in Health Sci Educ* **25**, 1057–1086 (2020). https://doi.org/10.1007/s10459-020-10009-8
	**State-of-the-art Review (N=0)**
	**Realist Reviews (N=0)**
	**Scoping Review (N=3)**
	Chong, J.Y., Ching, A.H., Renganathan, Y. *et al.* Enhancing mentoring experiences through e-mentoring: a systematic scoping review of e-mentoring programs between 2000 and 2017. *Adv in Health Sci Educ* **25**, 195–226 (2020). https://doi.org/10.1007/s10459-019-09883-8
	O'Brien, B.C., Battista, A. Situated learning theory in health professions education research: a scoping review. *Adv in Health Sci Educ* **25**, 483–509 (2020). https://doi.org/10.1007/s10459-019-09900-w
	Batt, A.M., Tavares, W. & Williams, B. The development of competency frameworks in healthcare professions: a scoping review. *Adv in Health Sci Educ* **25**, 913–987 (2020). https://doi.org/10.1007/s10459-019-09946-w
	**Meta-Ethnographic Reviews (N=0)**
	**Integrative Reviews (N=0)**
	**Perspectives on Medical Education**

	**Systematic Review (N=1)**
	Skjevik EP, Boudreau JD, Ringberg U, et al. Group mentorship for undergraduate medical students—a systematic review. *Perspectives on Medical Education*. 2020;9(5):272-280. https://doi.org/10.1007/s40037-020-00610-3
	**Narrative Review (N=0)**
	**Critical Review (N=0)**
	**State-of-the-art Review (N=0)**
	**Realist Reviews (N=0)**
	**Scoping Review (N=1)**
	Chan TM, Dzara K, Dimeo SP, Bhalerao A, Maggio LA. Social media in knowledge translation and education for physicians and trainees: a scoping review. *Perspectives on Medical Education*. 2019;9(1):20-30. https://doi.org/10.1007/s40037-019-00542-7
	**Meta-Ethnographic Reviews (N=0)**
	**Integrative Reviews (N=0)**
**2024**	**Medical Education**
	**Systematic Review (N=3)**
	Winderbaum J, Coventry LL. The benefits, barriers and facilitators of mentoring programs for first-year doctors: A systematic review. *Med Educ*. 2024; 58(6): 687-696. doi:10.1111/medu.15299
	Calo M, Judd B, Peiris C. Grit, resilience and growth-mindset interventions in health professional students: A systematic review and meta-analysis. *Med Educ*. 2024; 58(8): 902-919. doi:10.1111/medu.15391
	Lucas HC, Upperman JS, Robinson JR. A systematic review of large language models and their implications in medical education. *Med Educ*. 2024; 58(11): 1276-1285. doi:10.1111/medu.15402
	**Narrative Review (N=4)**
	Fruhstorfer BH, Jenkins SP, Davies DA, Griffiths F. International short-term placements in health professions education—A meta-narrative review. *Med Educ*. 2024; 58(7): 797-811. doi:10.1111/medu.15294
	Gomez K, Edwards HL, Kirby J. Livestreaming clinical experience to remotely located learners: A critical narrative review. *Med Educ*. 2024; 58(9): 1032-1041. doi:10.1111/medu.15392
	Edelist T, Friesen F, Ng S, et al. Critical reflection in team-based practice: A narrative review. *Med Educ*. 2024; 58(10): 1166-1177. doi:10.1111/medu.15462
	Lam JTH, Coret M, Khalil C, Butler K, Giroux RJ, Martimianakis Maria Athina (Tina). The need for critical and intersectional approaches to equity efforts in postgraduate medical education: A critical narrative review. *Med Educ*. 2024; 58(12): 1442-1461. doi:10.1111/medu.15425
	**Critical Review (N=2)**
	Orchard AR, Sitoh J, Wyatt A, Moore M. Music in medical education: A critical interpretive synthesis. *Med Educ*. 2024; 58(5): 507-522. doi:10.1111/medu.15255
	McKivett A, Paul D. Recreating the future—Indigenous research paradigms in health professional education research. *Med Educ*. 2024; 58(1): 149-156. doi:10.1111/medu.15154
	**State-of-the-art Review (N=0)**
	**Realist Reviews (N=0)**
	**Scoping Review (N=4)**
	Gutgeld-Dror M, Laor N, Karnieli-Miller O. Assertiveness in physicians' interpersonal professional encounters: A scoping review. *Med Educ*. 2024; 58(4): 392-404. doi:10.1111/medu.15222
	Kajee N, Montero-Marin J, Saunders KEA, Myall K, Harriss E, Kuyken W. Mindfulness training in healthcare professions: A scoping review of systematic reviews. *Med Educ*. 2024; 58(6): 671-686. doi:10.1111/medu.15293
	Yaros J, de Mortier C, oude Egbrink M, Evers S, Paulus A. Cost identification in health professions

	education: A scoping review. *Med Educ*. 2024; 58(8): 920-929. doi:10.1111/medu.15393
	Sarraf-Yazdi S, Pisupati A, Goh CK, et al. A scoping review and theory-informed conceptual model of professional identity formation in medical education. *Med Educ*. 2024; 58(10): 1151-1165. doi:10.1111/medu.15399
	**Meta-Ethnographic Reviews (N=0)**
	**Integrative Reviews (N=0)**
	**Academic Medicine**
	**Systematic Review (N=5)**
	Magnan, Elizabeth MD, PhD; Weyrich, Meghan MPH; Miller, Marykate MS; Melnikow, Joy MD, MPH; Moulin, Aimee MD, MAS; Servis, Mark MD; Chadha, Puja MD; Spivack, Sarah; Henry, Stephen G. MD. Stigma Against Patients With Substance Use Disorders Among Health Care Professionals and Trainees and Stigma-Reducing Interventions: A Systematic Review. Academic Medicine 99(2):p 221-231, February 2024. | DOI: 10.1097/ACM.0000000000005467
	Marhoffer, Elizabeth A. MD; Ein-Alshaeba, Samer MD; Grimshaw, Alyssa A. MBA, MSLIS; Holleck, Jürgen L. MD; Rudikoff, Benjamin; Bastian, Lori A. MD, MPH; Gunderson, Craig G. MD. Gender Disparity in Full Professor Rank Among Academic Physicians: A Systematic Review and Meta-Analysis. Academic Medicine 99(7):p 801-809, July 2024. | DOI: 10.1097/ACM.0000000000005695
	Lund, Sarah MD; Griffeth, Elaine M. MD; Williamson, Andrea MD; Collings, Amelia MD; Gudmundsdottir, Hallbera MD; Han, Amy MD; Kearse, LaDonna MD; Kratzke, Ian M. MD; Wilkins, Parvathi MBBS, PhD; Prokop, Larry J. MLS; Cook, David A. MD, MHPE. Gender Differences in Autonomy Granted to Residents and Fellows During Procedural Training: A Systematic Review and Meta-Analysis. Academic Medicine 99(8):p 922-930, August 2024. | DOI: 10.1097/ACM.0000000000005673
	Deshpande, Saarang R. MD; Lepore, Gina; Wieland, Lily; Kogan, Jennifer R. MD. Racial and Ethnic Bias in Letters of Recommendation in Academic Medicine: A Systematic Review. Academic Medicine 99(9):p 1032-1037, September 2024. | DOI: 10.1097/ACM.0000000000005688
	**Narrative Review (N=0)**
	**Critical Review (N=1)**
	Barker, Andrea M. MPAS, PA-C; Konopasky, Abigail W. PhD; Varpio, Lara PhD; Soh, Michael PhD; Poole, Brian R. MD; Stalmeijer, Renée E. MSc, PhD. How Advanced Practice Clinicians Support Education for and Contribute to Education of Graduate Medical Education Trainees: A Critical Literature Review. Academic Medicine 99(11):p 1286-1297, November 2024. | DOI: 10.1097/ACM.0000000000005858
	**State-of-the-art Review (N=0)**
	**Realist Reviews (N=0)**
	**Scoping Review (N=10)**
	Hanson, Mark D. MD, MEd; Pang, Celeste PhD; Springall, Elena MLIS; Kulasegaram, Kulamakan PhD; Eva, Kevin W. PhD. Patient Engagement in Medical Trainee Selection: A Scoping Review. Academic Medicine 99(1):p 98-105, January 2024. | DOI: 10.1097/ACM.0000000000005450
	Hamilton, Ailsa Lauren MBChB (Hons); Layden, Elizabeth A. MBChB, DLM; Storrar, Neill MBChB, MD; Skinner, Janet MBChB, MMedEd; Harden, Jeni MA, MPhil, PhD; Wood, Morwenna MA, MBBS, DPhil. Definition, Measurement, Precursors, and Outcomes of Trust Within Health Care Teams: A Scoping Review. Academic Medicine 99(1):p 106-117, January 2024. | DOI: 10.1097/ACM.0000000000005320

	Webber, Sarah MD; Semia, Sanaa; Nacht, Carrie L. MPH; Garcia, Sarahi; Kloster, Heidi MD; Vellardita, Lia MA; Kieren, Madeline Q.; Kelly, Michelle M. MD, PhD. Physician Work-Personal Intersection: A Scoping Review of Terms, Definitions, and Measures. Academic Medicine 99(3):p 331-339, March 2024. | DOI: 10.1097/ACM.0000000000005579
	Steinert, Yvonne PhD; Fontes, Kimberly MSc(A), RN; Mortaz-Hejri, Sara MD, PhD; Quaiattini, Andrea MA, MLIS; Yousefi Nooraie, Reza PhD, MD. Social Network Analysis in Undergraduate and Postgraduate Medical Education: A Scoping Review. Academic Medicine 99(4):p 452-465, April 2024. | DOI: 10.1097/ACM.0000000000005620
	Hansen, Allison; Klute, Ryan M. MS; Yadav, Manajyoti MD; Bansal, Saurabh MD; Bond, William F. MD. How Do Learners Receive Feedback on Note Writing? A Scoping Review. Academic Medicine 99(6):p 683-690, June 2024. | DOI: 10.1097/ACM.0000000000005653
	Hsu, Helen C.H. MSc; Martin, Tyler; Teunissen, Pim W. MD, PhD; Eva, Kevin W. PhD. Conceptualizing Educational Comparability in Distributed Health Professions Education: A Scoping Review. Academic Medicine 99(6):p 691-698, June 2024. | DOI: 10.1097/ACM.0000000000005679
	Romanova, Anna MD, MSc; Touchie, Claire MD, MHPE; Ruller, Sydney MSc; Kaka, Shaima MD; Moschella, Alexa; Zucker, Marc MD; Cole, Victoria MIS, MScN; Humphrey-Murto, Susan MD, MEd. Learning Plan Use in Undergraduate Medical Education: A Scoping Review. Academic Medicine 99(9):p 1038-1045, September 2024. | DOI: 10.1097/ACM.0000000000005781
	Visser, Eva H. MD; Oosterveld, Berdien MD; Slootweg, Irene A. PhD; Vos, Hedwig M.M. MD, PhD; Adriaanse, Marieke A. PhD; Schoones, Jan W. MA; Brakema, Evelyn A. MD, PhD. The Development and Characteristics of Planetary Health in Medical Education: A Scoping Review. Academic Medicine 99(10):p 1155-1166, October 2024. | DOI: 10.1097/ACM.0000000000005796
	Lindsley, Janet E. PhD; Abali, Emine E. PhD; Asare, Elliot A. MD, MS; Chow, Candace J. PhD, MA; Cluff, Caden; Hernandez, Marisol MLS, MA; Jamieson, Susan PhD, EdD; Kaushal, Amit MD; Woods, Nicole N. PhD. Contribution of Basic Science Education to the Professional Identity Development of Medical Learners: A Critical Scoping Review. Academic Medicine 99(11):p 1191-1198, November 2024. | DOI: 10.1097/ACM.0000000000005833
	Ismaeel, Romaisa; Pusic, Luka; Gottlieb, Michael MD; Chan, Teresa M. MD, MHPE; Oyedokun, Taofiq O. MBChB, MMed; Thoma, Brent MD, PhD. Bias in Observed Assessments in Medical Education: A Scoping Review. Academic Medicine 99(12):p 1438-1450, December 2024. | DOI: 10.1097/ACM.0000000000005794
	**Meta-Ethnographic Reviews (N=0)**
	**Integrative Reviews (N=1)**
	Smith SE, Livingston P, Carney E, Mardon J, Tallentire VR. Snakes and ladders: An integrative literature review of refugee doctors' workforce integration needs. *Med Educ*. 2024; 58(7): 782-796. doi:10.1111/medu.15290
	**Medical Teacher**
	**Systematic Review (N=2)**
	Fukui, N., Partain, D. K., Yeow, M. E., Farfour, H. N., Prokop, L., & Barwise, A. (2023). Learning to collaborate with medical interpreters in health professions education: A systematic review of training programs. *Medical Teacher*, *46*(2), 258–272. https://doi.org/10.1080/0142159X.2023.2249211
	Fonseca, M., Marvão, P., Oliveira, B., Heleno, B., Carreiro-Martins, P., Neuparth, N., & Rendas, A. (2023). The effectiveness of concept mapping as a tool for developing critical thinking in undergraduate medical education – a BEME systematic review: BEME Guide No. 81. *Medical Teacher*, *46*(9), 1120–1133. https://doi.org/10.1080/0142159X.2023.2281248

	**Narrative Review (N=3)**
	McGaghie, W. C., Barsuk, J. H., Wayne, D. B., & Issenberg, S. B. (2023). Powerful medical education improves health care quality and return on investment. *Medical Teacher*, *46*(1), 46–58. https://doi.org/10.1080/0142159X.2023.2276038
	Sidhu, N. S., & Fleming, S. (2023). Re-examining single-moment-in-time high-stakes examinations in specialist training: A critical narrative review. *Medical Teacher*, *46*(4), 528–536. https://doi.org/10.1080/0142159X.2023.2260081
	Sharma, A., Smyth, L., Jian, H., Vargas, N., Bowles, D., & Hunter, A. (2023). Are we teaching the health impacts of climate change in a clinically relevant way? A systematic narrative review of biomechanism-focused climate change learning outcomes in medical curricula. *Medical Teacher*, *46*(3), 414–422. https://doi.org/10.1080/0142159X.2023.2256963
	**Critical Review (N=0)**
	**State-of-the-art Review (N=0)**
	**Realist Reviews (N=0)**
	**Scoping Review (N=6)**
	Sorgini, A., Istl, A. C., Downie, M. L., & Kirpalani, A. (2023). Pride & prejudice: A scoping review of LGBTQ + medical trainee experiences. *Medical Teacher*, *46*(1), 73–81. https://doi.org/10.1080/0142159X.2023.2229503
	Li, H., Upreti, T., Do, V., Dance, E., Lewis, M., Jacobson, R., & Goldberg, A. (2023). Measuring wellbeing: A scoping review of metrics and studies measuring medical student wellbeing across multiple timepoints. *Medical Teacher*, *46*(1), 82–101. https://doi.org/10.1080/0142159X.2023.2231625
	Doyle, A. J., Sullivan, C., O'Toole, M., Tjin, A., Simiceva, A., Collins, N., Eppich, W. (2023). Training simulated participants for role portrayal and feedback practices in communication skills training: A BEME scoping review: BEME Guide No. 86 . *Medical Teacher*, *46*(2), 162–178. https://doi.org/10.1080/0142159X.2023.2241621
	Gordon, M., Daniel, M., Ajiboye, A., Uraiby, H., Xu, N. Y., Bartlett, R., Thammasitboon, S. (2024). A scoping review of artificial intelligence in medical education: BEME Guide No. 84. *Medical Teacher*, *46*(4), 446–470. https://doi.org/10.1080/0142159X.2024.2314198
	Beattie, J., Binder, M., & Fuller, L. (2023). Rural longitudinal integrated clerkships and medical workforce outcomes: A scoping review. *Medical Teacher*, *46*(4), 545–555. https://doi.org/10.1080/0142159X.2023.2260082
	Krebs, C. L., Thestrup, J., Hybschmann, J., Graber, K., Gjærde, L. K., Topperzer, M. K., Sørensen, J. L. (2023). A BEME review of educational programmes on the use of play in paediatric practice: BEME Guide No. 82. *Medical Teacher*, *46*(11), 1393–1403. https://doi.org/10.1080/0142159X.2023.2287983
	**Meta-Ethnographic Reviews (N=0)**
	**Integrative Reviews (N=0)**
	**Advances in Health Science Education**
	**Systematic Review (N=4)**
	Kaisti, I., Kulmala, P., Hintsanen, M. *et al.* The effects of mindfulness-based interventions in medical students: a systematic review. *Adv in Health Sci Educ* **29**, 245–271 (2024). https://doi.org/10.1007/s10459-023-10231-0
	Jensen, R.A.A., Musaeus, P. & Pedersen, K. Virtual patients in undergraduate psychiatry education: a systematic review and synthesis. *Adv in Health Sci Educ* **29**, 329–347 (2024). https://doi.org/10.1007/s10459-023-10247-6
	Trumble, E., Lodge, J., Mandrusiak, A. *et al.* Systematic review of distributed practice and retrieval practice in health professions education. *Adv in Health Sci Educ* **29**, 689–714 (2024). https://doi.org/10.1007/s10459-023-10274-3
	Aster, A., Laupichler, M.C., Zimmer, S. *et al.* Game design elements of serious games in the education of medical and healthcare professions: a mixed-methods systematic review of underlying theories and teaching effectiveness. *Adv in Health Sci Educ* **29**, 1825–1848 (2024). https://doi.org/10.1007/s10459-024-10327-1
	**Narrative Review (N=1)**

	Tan, E., Frambach, J., Driessen, E. *et al.* Opening the black box of school-wide student wellbeing programmes: a critical narrative review informed by activity theory. *Adv in Health Sci Educ* **29**, 663–687 (2024). https://doi.org/10.1007/s10459-023-10261-8
	**Critical Review (N=1)**
	Kalocsai, C., Agrawal, S., de Bie, L. *et al.* Power to the people? A co-produced critical review of service user involvement in mental health professions education. *Adv in Health Sci Educ* **29**, 273–300 (2024). https://doi.org/10.1007/s10459-023-10240-z
	**State-of-the-art Review (N=1)**
	Chan, C.A., Windish, D.M., Spak, J.M. *et al.* State-of-the-art review of medical improvisation curricula to teach health professional learners communication. *Adv in Health Sci Educ* **29**, 1025–1046 (2024). https://doi.org/10.1007/s10459-023-10296-x
	**Realist Reviews (N=0)**
	**Scoping Review (N=4)**
	Milgate, W., Copley, J. & Hill, J. Failing professional practice placements in allied health: What do we understand about the student experience? A scoping review. *Adv in Health Sci Educ* **29**, 301–327 (2024). https://doi.org/10.1007/s10459-023-10243-w
	Giroux, C.M., Kim, S., Sikora, L. *et al.* Social media as a mechanism of dissemination and knowledge translation among health professions educators: a scoping review. *Adv in Health Sci Educ* **29**, 993–1023 (2024). https://doi.org/10.1007/s10459-023-10294-z
	Medina-Córdoba, M., Cadavid, S., Espinosa-Aranzales, AF. *et al.* The effect of interprofessional education on the work environment of health professionals: a scoping review. *Adv in Health Sci Educ* **29**, 1463–1480 (2024). https://doi.org/10.1007/s10459-023-10300-4
	Mukhalalati, B., Yakti, O. & Elshami, S. A scoping review of the questionnaires used for the assessment of the perception of undergraduate students of the learning environment in healthcare professions education programs. *Adv in Health Sci Educ* **29**, 1501–1538 (2024). https://doi.org/10.1007/s10459-024-10319-1
	**Meta-Ethnographic Reviews (N=0)**
	**Integrative Reviews (N=1)**
	Merkebu, J., Veen, M., Hosseini, S. *et al.* The case for metacognitive reflection: a theory integrative review with implications for medical education. *Adv in Health Sci Educ* **29**, 1481–1500 (2024). https://doi.org/10.1007/s10459-023-10310-2
	**Perspectives on Medical Education**
	**Systematic Review (N=0)**
	**Narrative Review (N=1)**
	Snow Wangding, Lingard L, Haidet P, Vipler B, Javeed Sukhera, Moniz T. Disorienting or Transforming? Using the Arts and Humanities to Foster Social Advocacy. *Perspectives on Medical Education*. 2024;13(1):192-200. doi:https://doi.org/10.5334/pme.1213
	**Critical Review (N=0)**
	**State-of-the-art Review (N=0)**
	**Realist Reviews (N=0)**
	**Scoping Review (N=1)**
	Qian Hui Chew, Ethan Jian-Hui Maniam, Sim K. Inter-Professional Education Interventions, and Practice Outcomes Related to Healthcare Setting and Patients Within Mental Healthcare: A Scoping Review. *Perspectives on medical education*. 2024;13(1). doi:https://doi.org/10.5334/pme.1084
	**Meta-Ethnographic Reviews (N=0)**
	**Integrative Reviews (N=1)**
	Melro CM, Matheson K, Bombay A. What Outcomes Are Associated with Learning About Colonialism and Its Impacts on Indigenous Peoples in Health Professional Programs? A Critical Integrative Review. *Perspectives on Medical Education*. 2024;13(1):677-683. doi:https://doi.org/10.5334/pme.1407
